# Case Report: *FBN1* mutation screening in South African patients with Marfan syndrome

**DOI:** 10.3389/fgene.2025.1612411

**Published:** 2025-07-02

**Authors:** F. Mhlongo, C. Feben, A. Krause, N. Carstens

**Affiliations:** ^1^ Division of Human Genetics, National Health Laboratory Service & School of Pathology, Faculty of Health Sciences, The University of the Witwatersrand, Johannesburg, South Africa; ^2^ Genomics Centre, South African Medical Research Council, Cape Town, South Africa

**Keywords:** Marfan syndrome, understudied populations, molecular confirmation, FBN1 gene, genetic diagnostics in Africa, case series

## Abstract

Marfan syndrome (MFS) is a systemic heritable connective tissue disorder caused by pathogenic variants in the *FBN1* gene. Previous studies have documented the clinical utility of *FBN1* mutation screening as some nucleotide changes and functional domains are associated with specific clinical presentations, many of which are age dependent. However, molecular testing has not been incorporated into routine clinical service for MFS in South Africa. Here we present clinical phenotypes and molecular confirmation of MFS in a cohort of South African patients. Mutation screening using a targeted next-generation sequencing (NGS) panel identified seven heterozygous likely pathogenic and/or pathogenic *FBN1* variants in eleven South African patients with MFS. Two of these variants are novel. This study thus contributes to the description of the mutation spectrum of MFS in Africa and highlights the diagnostic utility and importance of *FBN1*-based mutation testing, especially in children and for prognostic purposes.

## Introduction

Marfan syndrome (MFS; OMIM #154700) is an autosomal dominant systemic disorder that affects connective tissue and falls under the broader group of heritable connective tissue disorders (HCTDs). The condition arises due to harmful variants in the *FBN1* gene (OMIM #134797), situated on chromosome 15q21.1. This gene spans a genomic region of approximately 237 kilobases and contains 65 exons. It encodes the fibrillin-1 protein, which is rich in cysteine and plays a crucial role in assembling microfibrils within the extracellular matrix of connective tissues. Mutations in *FBN1* can result in a significant decrease in microfibril formation (Dietz, 2017), disruption of the normal architecture of microfibrils (Chandra and Charteris, 2014), or elevated TGFβ signaling in the aortic wall (Gordon and Blobe, 2008). These molecular disruptions often present clinically as aortic root enlargement, mitral or tricuspid valve prolapse, dislocation of the ocular lens (ectopia lentis), and nearsightedness (myopia). Skeletal features commonly observed include excessive height, elongated limbs (dolichostenomelia), scoliosis, inward or outward chest deformities (pectus excavatum or carinatum), and protrusio acetabuli (Dietz, 2017).

As noted by De Maio et al. (2016), musculoskeletal signs play a key role in the diagnosis of MFS, even when symptoms may appear subtle—such as mild hip discomfort. These skeletal abnormalities can lead to significant joint issues and long-term disability. For instance, longstanding protrusio acetabuli has been linked to early-onset hip osteoarthritis (De Maio et al., 2016), while joint instability, especially in the knees, may compromise mobility and, in severe cases, result in reliance on mobility aids like wheelchairs (Kaissi et al., 2013). Despite these manifestations, most fatalities in MFS are due to cardiovascular complications. The global prevalence is estimated to be between 1 in 5,000 and 1 in 10,000 individuals (Dietz, 2017). Based on this estimate, approximately 10,000 individuals in South Africa may be living with MFS (Child et al., 2007).

Clinical diagnosis typically relies on the revised Ghent criteria (Loeys et al., 2010), which incorporate a range of phenotypic features. However, the utility of these criteria can be limited due to overlapping clinical characteristics with other HCTDs and the age-related variability in how MFS features appear. Since clinical management and associated risks vary between different connective tissue disorders (Loeys et al., 2010), establishing a precise diagnosis is essential. The updated Ghent nosology therefore emphasizes the importance of molecular testing to support early and accurate diagnosis (Loeys et al., 2010). Unfortunately, access to genetic testing remains limited in many low- and middle-income countries, including South Africa, where public health systems often lack the infrastructure and funding to support molecular diagnostics (Kamp et al., 2021). In this context, we present the utility of a targeted next-generation sequencing (NGS) gene panel to confirm MFS in a group of 14 individuals from 10 unrelated South African families.

## Methods

Following the acquisition of informed consent and, where applicable, assent, blood samples were collected from fourteen participants representing ten families, each with at least one member either clinically diagnosed with or suspected of having Marfan syndrome (MFS). MFS clinical diagnoses were made using the revised Ghent criteria (Loeys et al., 2010). All individuals had previously been evaluated at a Genetics Clinic, and relevant clinical information was extracted from their medical records. These data included the MFS systemic score, echocardiographic and ophthalmologic findings, and additional indicators suggestive of a heritable connective tissue disorder (HCTD). In some instances, assessments included a Beighton score to evaluate joint hypermobility, inspection of the uvula, and other specialized diagnostic evaluations. Due to limited access to magnetic resonance imaging in South Africa, assessments for features such as dural ectasia or hip abnormalities were not routinely performed, and conclusions regarding these findings cannot be drawn.

Genomic DNA was isolated from peripheral blood using the FlexiGene DNA kit (Qiagen, Hilden, Germany), following the manufacturer’s protocol. DNA library construction and template preparation were automated using the Ion Chef system (Thermo Fisher Scientific, Waltham, Massachusetts, USA). Sequencing was conducted on the Ion GeneStudio S5 System (Thermo Fisher Scientific). The sequencing panel included a set of genes known to be associated with MFS and related HCTDs, specifically *ACTA2*, *CBS*, *COL1A1*, *COL3A1*, *FBN1*, *FBN2*, *MYH11*, *SKI*, *SMAD2*, *SMAD3*, *TGFβ3*, *TGFβR1*, and *TGFβR2*. Read alignment to the GRCh37/hg19 human genome reference and variant calling were carried out using the Ion Torrent Suite Software version 5.14 with built-in plug-ins.

Variant annotation was performed using Ion Reporter Software version 5.14.1.0 (Thermo Fisher Scientific) along with the Ensembl Variant Effect Predictor (McLaren et al., 2016). For the FBN1 gene, interpretation was based on the reference transcript NM_000138.4. Variants were classified using the ACMG/AMP guidelines (Richards et al., 2015), as well as FBN1-specific recommendations described by Muiño-Mosquera et al. (2018) and Baudhuin et al. (2019). It is widely accepted that clinically relevant variants identified through NGS should be confirmed using Sanger sequencing—the traditional gold standard for DNA sequencing—due to its high accuracy (Arteche-López et al., 2021). Although no formal international guidelines universally mandate this, Sanger confirmation remains common practice for quality assurance, facilitating accurate segregation analysis and reliable clinical reporting. Ethical approval for the study was obtained from the Human Research Ethics Committee (Medical) of the University of the Witwatersrand (clearance number M191184).

## Results

**FIGURE 1:**
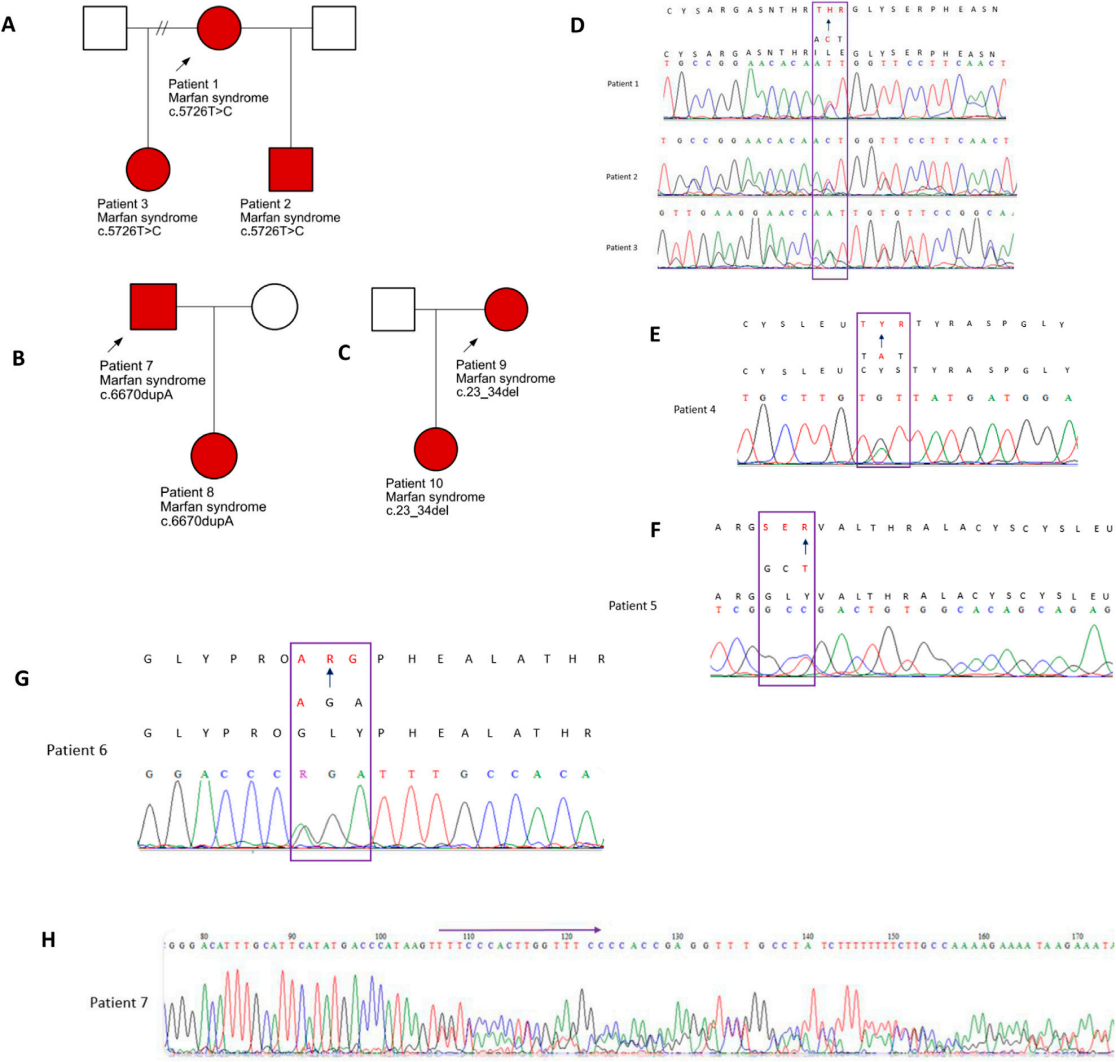
Family pedigrees: affected individuals are indicated by the red shading **(A)**. Pedigree for patients 1–3. **(B)** Pedigree for patients 7 and 8 and **(C)** for patients 9 and 10. **(D–H)** Electropherograms of the five variants validated by Sanger sequencing and identified in patients 1 to 8.

Seven *FBN1* variants classified as either likely pathogenic or pathogenic were detected in eleven individuals from seven separate families. These individuals had either a confirmed clinical diagnosis of MFS or presented with features indicative of the disorder. The identification of these variants provided molecular confirmation of the suspected diagnoses (Table 1). Among the seven identified variants, five were missense changes, one was a frameshift mutation, and one involved an in-frame deletion. Five of these had been previously documented in the literature, whereas two were novel findings. Electropherograms from Sanger sequencing are presented in Figure 1 (patients 1–8). Unfortunately, two variants (c.478T>C and c.23_34del) could not be validated through Sanger sequencing due to sample depletion following next-generation sequencing.

**TABLE 1:** The *FBN1* (likely) pathogenic variants identified in this study and the clinical descriptions of the patients.

Family	Patient	Ethnic group	Sex	Age in years	Clinical				Fulfils ghent (at initial clinic visit)	*FBN1* (likely) pathogenic variant	Exon	ClinVar accession number	Sanger validation	First report
					Cardiac	Ocular	Systemic Score	Components						
Family 1	Patient 1^a^	A	M	34			7	PC(1), HD(1), SS(1), WS+TS(3), FF (1)	Y EL, SS	c.5726T>C (p.Ile1909Thr) Heterozygous	47	VCV000200189.33	Y	Loeys et al. (2001) ^b^
Family 1	Patient 2	A	M	12	AD	EL, M	9	WS + TS (3), PC(1), PP(1), S(1), FF (1), SS(1), M(1)	Y AD, EL, FH	c.5726T>C (p.Ile1909Thr) Heterozygous	47	VCV000200189.33	Y	Loeys et al. (2001) ^b^
Family 1	Patient 3	A	F	7	AD	EL	9	WS + TS (3), PC(1), PP(1), S(1), FF (1), M(1), MVP(1)	Y AD, EL, FH	c.5726T>C (p.Ile1909Thr) Heterozygous	47	VCV000200189.33	Y	Loeys et al. (2001) ^b^
Family 2	Patient 4	A	F	37		EL	6	WS + TS (3), US:LS (1), FF(1), MVP(1)	N EL	c.3794G>A (p.Cys1265Tyr) Heterozygous	30	VCV002137697.5	Y	Attanasio et al. (2008) ^b^
Family 3	Patient 5	A	F	11	AD	EL	4	TS (1), PP(1), M(1) MVP(1)	Y AD, EL	c.640G>A (p.Gly214Ser) Heterozygous	7	VCV000199956.32	Y	Collod-Beroud et al. (2003) ^b^
Family 4	Patient 6	A	M	9	AD, MVPR	EL, M	6	PE(1), PP(1), US:LS (1), FF(1), M (1), MVP(1)	Y AD,EL	c.3037G>A (p.Gly1013Arg) Heterozygous	25	VCV000177648.41	Y	Nijbroek et al. (1995) ^b^
Family 5	Patient 7^a^	MA	M	32	AA		5	TS(1), US:LS(1), S(1), FF(1) M (1)	N AD	c.6670dupA (p.Thr2224AsnfsTer6) Heterozygous	55	VCV002579125.1	Y	This study
Family 5	Patient 8	MA	F	6	MVP		2	US:LS(1), MVP (1)	N	c.6670dupA (p.Thr2224AsnfsTer6) Heterozygous	55	VCV002579125.1	Y	This study
Family 6	Patient 9^a^	A	F	33	MVP		7	EE(1), SS(1), TS/WS(3), US:LS(1), HD(1), MVP(1)	Y FH, SS	c.23_34del (p.Glu8_Leu11del) Heterozygous	2	VCV002579132.1	ND	This study
Family 6	Patient 10	A	F	5			2	US:LS (1), FF(1)	N FH	c.23_34del (p.Glu8_Leu11del) Heterozygous	2	VCV002579132.1	ND	This study
Family 7	Patient 11	A	F	11		EL	4	WS(1), EE(1), FF(1) SS(1)	N EL	c.478T>C (p.Cys160Arg) Heterozygous	5	VCV000374203.13	ND	Comeglio et al. (2007) ^b^

^a^ Parent in the family, A, African; AA, Aortic aneurysm; AD, Aortic root dilatation (Z>2); EL, Ectopia lentis; FH, family history; ND, Not done (DNA depleted); Y, Yes. Systemic score, used in the revised Ghent criteria for MFS by Loeys et al. (2010): EE, reduced elbow extension; FF, facial features; HD, hindfoot deformity; M, myopia; MA, mixed ancestry; MVP, mitral valve prolapse; MVPR, mitral valve prolapse with regurgitation; PC, Pectus carinatum; PE, Pectus excavatum; PP,pes planus; S, scoliosis; SS, skin striae; TS, Thumb sign; US:LS, reduced upper segment: lower segment and increased arm span; WS, Wrist sign.

^b^ Previously reported pathogenic variant either in and/or by Tiecke et al. (2001), Biggin et al. (2004), Loeys et al. (2004), Rommel et al. (2005), Baetens et al. (2011), Dong et al. (2012), Wang et al. (2013), Yang et al. (2016), Madar et al. (2019) or Renner et al. (2019). The blank spaces indicate the absence of clinical features in the patient at the time of the clinical visit.

A number of the detected variants fell within exons 24 to 32 of the *FBN1* gene, a genomic region often referred to as the “neonatal region” due to its known association with the more severe, early-onset neonatal form of MFS (Franken, 2016). Variants within this region were found in Patient 4 (c.3794G>A, p.Cys1265Tyr) and Patient 6 (c.3037G>A, p.Gly1013Arg). The structural integrity of fibrillin-1 is highly dependent on cysteine residues, and substitutions that eliminate these residues are linked to more serious cardiovascular outcomes, particularly involving the aorta (Baudhuin et al., 2019; Stengl et al., 2020). Specifically, the c.3037G>A variant has been noted as a minor hotspot for severe, early-onset symptoms (Madar et al., 2019). In addition, individuals carrying FBN1 frameshift mutations are also thought to face a greater risk of developing aortic complications (Baudhuin et al., 2015).

In our study group, three of the fourteen individuals clinically suspected of having MFS did not have any detectable pathogenic or likely pathogenic variants in the *FBN1* gene. None of these three patients met the diagnostic criteria outlined in the revised Ghent nosology. However, each presented with features that raised a strong clinical suspicion of MFS, highlighting the challenges of clinical diagnosis in the absence of definitive genetic or systemic criteria. Patient 12, a 16-year-old male of Caucasian descent, had tall stature, myopia, and a systemic score of 7, just below the threshold for a definitive diagnosis. Patient 13, a 22-year-old African male, presented with a lower systemic score of 4 but had mild dysmorphic features and a family history of sudden unexplained death, prompting concern for an underlying connective tissue disorder. Patient 14, a 12-year-old African female, showed a combination of suggestive features including a positive wrist-thumb sign, joint laxity, arachnodactyly, mild scoliosis, and a systemic score of 7. Although her family history was not definitive for MFS, multiple family members were noted to be tall, and she also had an unspecified hip abnormality. While the available clinical data were insufficient to meet the revised Ghent criteria or to definitively diagnose MFS, these cases illustrate the diagnostic ambiguity that can arise in practice and reinforce the importance of comprehensive molecular testing to support differential diagnosis.

## Discussion

In 1998 Stuart and Wilson argued that an early MFS diagnosis enables optimal clinical management and a significant improvement in long-term outcomes (Stuart and Wilson, 1998). Modern sequencing techniques and “exome first” diagnostic strategies have enabled and demonstrated this in a number of countries. However, African families affected by MFS are still not benefitting from this 24 years later.

Routine monitoring of individuals with Marfan syndrome (MFS) typically includes annual eye exams and echocardiographic assessments. However, in the South African context, echocardiography poses significant challenges due to its high cost and limited accessibility to specialist healthcare services. As a result, regular follow-up using this imaging modality is often not feasible. A confirmed diagnosis of MFS can enable early medical intervention, which may reduce the risk of severe complications associated with the condition (Stengl et al., 2020). In resource-constrained settings like South Africa, this highlights the importance of timely molecular diagnosis, which could reduce reliance on repeated clinical screening methods such as echocardiograms (Child et al., 2007). Moreover, once a molecular diagnosis is made, at-risk relatives can be offered predictive or prenatal testing where appropriate.

Molecular confirmation is particularly valuable in individuals who do not yet meet the clinical diagnostic thresholds defined by the revised Ghent criteria—especially pediatric patients. For example, Patient 8 displayed suggestive, but inconclusive, features of MFS, while her father (Patient 7) had already undergone surgery for an aortic aneurysm prior to receiving a molecular diagnosis. Another case, Patient 9, had a systemic score exceeding 7 at presentation, along with a family history that pointed toward MFS, although the details were not well documented. Molecular testing in her case was essential for clarifying the diagnosis and guiding her clinical management. Furthermore, her young child—who did not initially meet clinical diagnostic criteria—tested positive for the familial variant, enabling clinicians to implement an early, proactive management plan.

Molecular diagnostic testing for MFS is not currently available to patients in the South African State healthcare system. Our study is the one of very few molecular studies in sub-Saharan Africa to perform mutation screening on MFS patients. To the best of our knowledge, the only other MFS molecular report in South Africa is by Child et al. (2007). In accordance with the literature, we acknowledge several benefits of molecular diagnostic testing and infer that mutation screening of the *FBN1* gene is an appropriate diagnostic approach in the South African patients with a phenotype suggestive of MFS. MFS is currently diagnosed in the South African State healthcare system using clinical assessments alone. However, it is uncertain if the diagnostic criteria are universally applicable to patients of African ancestry (Child et al., 2007) and a large portion of South Africans struggle to get access to the specialists needed to make a reliable MFS diagnosis. This is further compounded by the fact that a definitive MFS diagnosis is even more difficult to reach in younger individuals due to the age-dependent physical manifestations of MFS.

In our cohort, we observed that some individuals with confirmed *FBN1* pathogenic variants did not fulfill the revised Ghent criteria at the time of assessment, likely due to incomplete clinical workup or age-related phenotypic expression. For instance, Patient 7 had a systemic score of 5 but lacked imaging that may have revealed additional diagnostic features such as dural ectasia or hip dysplasia. Similarly, Patient 4 had a systemic score of 6, also without imaging and no ectopia lentis—placing him close to, but not over, the diagnostic threshold. These examples underscore the limitations of applying clinical criteria in isolation, particularly in settings where access to imaging is limited, and among pediatric or young adult patients where key features may be age-dependent. This reinforces the utility of molecular testing not only as a confirmatory tool but also as a primary component of the diagnostic workflow for suspected MFS in low-resource settings.

The identification of *FBN1* pathogenic variants confirms an MFS diagnosis, streamlines clinical care, provides information on prognosis and possible complications, thereby improving the life expectancy and -quality of affected individuals. Confirmation of diagnosis is further important to target appropriate surveillance and minimise unnecessary expenditure on an array of inappropriate screening/diagnostic tests. This is important for resource allocation in the South African State healthcare system, which is the primary healthcare provider for 80% of the South African population. Additionally, affected individuals and their family members may benefit from prenatal diagnosis, pre-symptomatic and predictive testing. Management of MFS, particularly cardiovascular complications in affected individuals using hemodynamic stress-reducing agents such as β-blockers and Angiotensin-converting enzyme inhibitors has proven to be more efficient in preventing aortic dissection if started early (Dietz, 2017). Thus, additional benefits of an early diagnosis include pre-emptive management of aortic complications, which are primarily the cause of MFS-related morbidities and mortalities. Furthermore, the efficacy of the treatment used for individuals with stand-alone osteoarthritis and those with osteoarthritis caused by MFS for example, may vary based on the treatment choice. As a result, the lack of the knowledge of the etiology of the joint disease (osteoarthritis) may result in management using a symptom-based option for MFS-related osteoarthritis instead of managing the root problem (Kaissi et al., 2013).

A molecular diagnosis is further significant because the type and location of a mutation have been reported to have an effect on the severity of a phenotype. For example, knowledge of the particular *FBN1* pathogenic variant will significantly impact the genetic counselling and clinical management of families 2, 4 and 5 in this study. Individuals with *FBN1* mutations in the neonatal region are predisposed to a severe phenotype and those with frameshift mutations have been described to have an increased risk of aortic complications (Baudhuin et al., 2015), necessitating specialist cardiac follow up. Thus *FBN1* testing will aid in the identification of individuals who should be more closely monitored for aortic complications, thereby enhancing personalized patient care. This can in turn improve the quality of life of MFS-affected individuals.

## Conclusion

Early identification of pathogenic *FBN1* variants enables genetic counselling, streamlines clinical care, and provides information on prognosis and possible complications, thereby improving the life expectancy and quality of affected individuals. Based on our findings, we advocate for the inclusion of *FBN1* genetic testing in the diagnostic workup of children presenting with features indicative of Marfan syndrome.

Our study highlights the impact of an early molecular MFS diagnosis on diagnostic spending, genetic counselling and clinical management in a low resource setting. We furthermore contribute two novel pathogenic FBN1 variants to the mutation spectrum of MFS.

Given the absence of identified *FBN1* variants in three individuals in this study, we plan to conduct more detailed phenotypic assessments along with broader genetic investigations, including exome sequencing and analysis for potential copy number variants, to further explore the underlying cause of their clinical features.

## Data Availability

The variants described here were submitted to ClinVar and can be accessed using the accession numbers provided in Table 1 (Organization ID 508172). Data available from corresponding author upon reasonable request.
